# A Pigmented Lesion of the Vulva Revealing Aggressive Melanoma: A Case Report

**DOI:** 10.7759/cureus.60257

**Published:** 2024-05-14

**Authors:** Soumiya Samba, El Ouardani Soufia, Tariq Bouhout, Ouissam Al Jarroudi, Soufiane Berhili, Mohamed Moukhlissi, Sami Aziz Brahmi, Badr Serji, Said Afqir, Loubna Mezouar

**Affiliations:** 1 Department of Radiation Oncology, Mohammed VI University Hospital, Faculty of Medicine and Pharmacy, Mohammed First University, Oujda, MAR; 2 Department of Oncology, Mohammed VI University Hospital, Faculty of Medicine and Pharmacy, Mohammed First University, Oujda, MAR; 3 Department of Surgical Oncology, Mohammed VI University Hospital, Faculty of Medicine and Pharmacy, Mohammed First University, Oujda, MAR

**Keywords:** chemotherapy, radiation therapy, melanoma, vulvar mass, vulva

## Abstract

Vulvar melanoma (VM) is a rare and aggressive malignancy presenting unique challenges in diagnosis and management. This report presents the case of a 61-year-old female patient and explores the clinical characteristics, diagnostic modalities, treatment strategies, and prognosis associated with VM. The patient presented with a painless mass on the labia majora, which turned out to be an undifferentiated malignant tumor process consistent with melanoma on examination. Immunohistochemical analysis confirmed the diagnosis and subsequent imaging revealed metastatic disease necessitating palliative chemotherapy following radiotherapy. VM is a rare and aggressive form of melanoma. While surgery is the standard of care for early stages, advanced stages require a combination of immunotherapy and targeted treatments. Clinical trials are vital to improve our understanding of this condition and the various aspects of its care. Collaboration among experts is essential to achieve progress in managing these patients.

## Introduction

Mucosal melanomas (MM) are rare tumors, and these are less frequently encountered compared to cutaneous melanomas (CM) [1]. According to the American Cancer Society's cancer statistics, melanomas accounted for 5% of all cancers in the United States in 2023, of which ≤2% were MM [2]. MMs originate from melanocytes in the mucous membranes of the body [3], with approximately 50% located in the head and neck, 37% in the gastrointestinal tract, 6% in the genital tract in women, and 4% in the urinary tract [1]. Despite similarities in diagnostic and therapeutic aspects, MM and CM differ in their clinical presentations, molecular profile, treatment response, and prognosis, emphasizing the necessity to differentiate between the entities [3].

This report discusses an uncommon variant of MM in women, namely vulvar melanoma (VM), which represents approximately 1% of all melanomas [4]. Its positive diagnosis is based on histopathological and immunohistochemical examination. Classically, VM expresses markers such as SOX10 [SRY (sex-determining region of Y)-Box 10], S100 protein, Melan A, and HMB45 (human melanoma black 45) [5]. Molecularly, VM is characterized by a high frequency of c-KIT mutation (around 27%) and fewer BRAF mutations (around 8.2%) compared to CM, which harbors 55-60% BRAF mutations [6]. The treatment primarily depends on the stage of the disease. Surgery with negative resection margins is the standard treatment for localized VM. For metastatic disease, the treatment is similar to CM and is primarily based on immunotherapy and targeted therapies in cases of targetable mutations [7].

VM remains an aggressive tumor with a poor prognosis despite therapeutic advances, with an overall five-year survival estimated at 47% for all stages combined, compared to 92% for CM [4]. We present this case to share our diagnostic experience involving a vulvar mass in a 61-year-old female patient, which was later found to be melanoma.

## Case presentation

**Figure 1:**
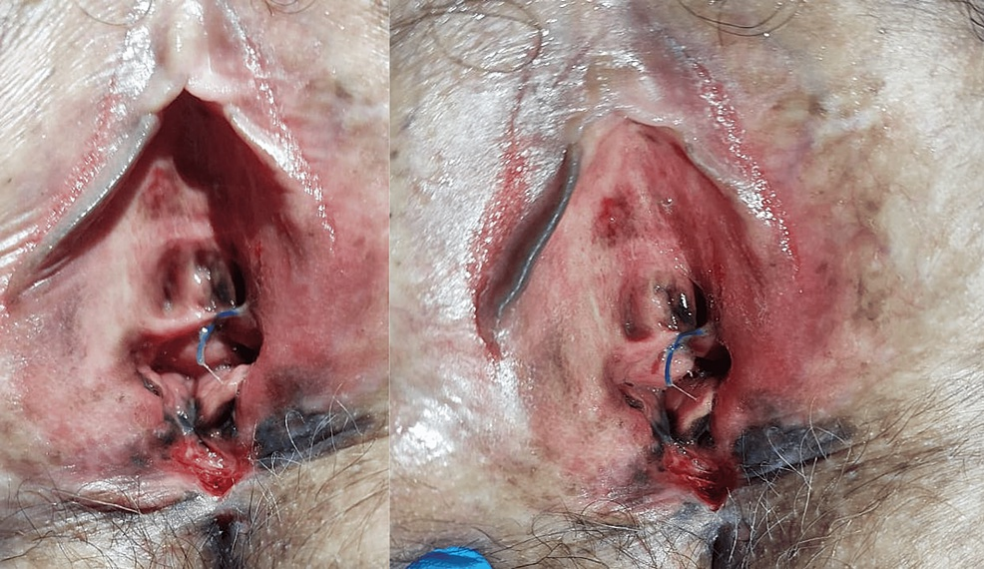
Clinical image showing multifocal pigmented and asymmetric lesions with irregular dark borders in the vulva

The patient was a 61-year-old female with no significant medical history. Her disease history dated back six months with the appearance of a mass on the labia majora, with heterogeneous black, brown spots, painless, which had gradually increased in volume and started to bleed, all evolving with the preserved general condition. Upon initial examination, the patient was conscious and apyretic. The gynecological examination revealed an irregular pigmented mass at the separation of the labia minora, infiltrating the vulva and urethral meatus (Figure 1). Vaginal examination showed a normal cervix without anomalies, with free vaginal fornices and walls. Examination of the inguinal and supraclavicular lymph nodes was unremarkable.

**Figure 2:**
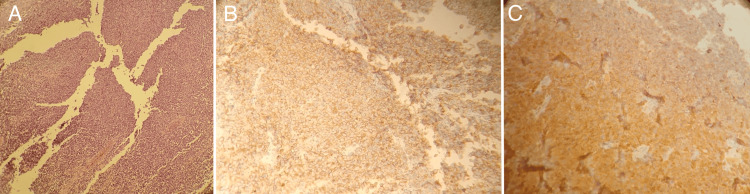
Histological examination of the vulvar pigmented lesion A: Low power view showing vulvar mucosa lined by squamous epithelium. The underlying lamina propria is invaded by diffuse sheets of malignant tumoral cells. B: Tumoral cells showing positive cytoplasmic staining for HMB45. C: Cytoplasmic expression of PS100 by tumoral cells

Subsequently, the patient underwent a biopsy of one pigmented lesion of the vulva, which revealed an undifferentiated malignant tumor process. Immunohistochemical analysis favored a malignant melanoma with positive staining for anti-PS100 and anti-HMB45 antibodies, and absence of staining for anti-AE1/AE3, anti-CD45, anti-desmin, and anti-myogenin antibodies (Figure 2)

**Figure 3:**
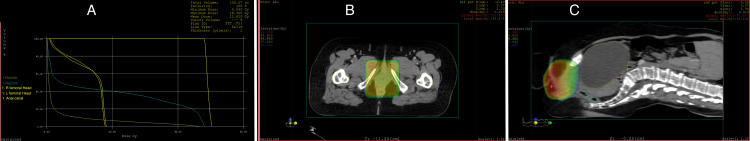
Radiotherapy A: Radiotherapy was administered in recommended doses for the organs at risk. B: Axial section. B: Sagittal section: dosimetric assessment of radiotherapy colorwash dose coverage of vulva primary tumor PTV PTV: planning target volume

A staging scan comprising contrast-enhanced brain, cervical, thoracic, abdominal, and pelvic CT scan did not reveal any secondary localization. The case was then reviewed by the surgery team and they ruled out any operative treatment. The patient underwent exclusive hypofractionated three-dimensional conformal external beam radiotherapy targeting the mass with a total dose of 48 Gy delivered in 16 fractions of 3 Gy over four days every seven days. The radiotherapy was administered following the recommended doses for the organs at risk (Figure 3).

**Figure 4:**
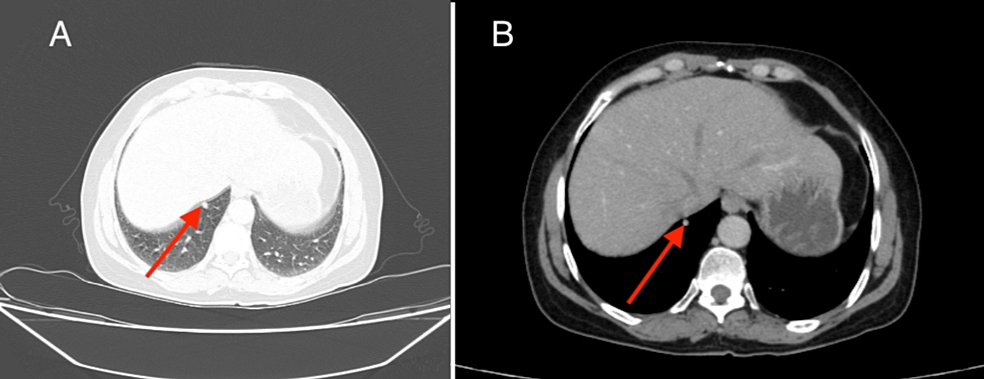
Axial chest CT scan (A) Lung window and (B) mediastinal window demonstrating an 8 mm pulmonary nodule at the right basal level CT: computed tomography

A month after the completion of radiation therapy, a follow-up chest scan showed a progression at the pulmonary level with an appearance of an 8 mm pulmonary nodule at the right basal level (Figure 4), leading to the initiation of palliative chemotherapy with doxorubicin 1000 mg/m² on days 1 and 21.

Radiotherapy was delivered without any acute side effects; the patient is currently undergoing palliative chemotherapy and her disease course has been stable.

## Discussion

Melanoma is the sixth most common cancer in the United States according to the American Cancer Society's cancer statistics in 2023 [2]. VM constitutes a rare localization of melanoma, representing less than 1% of melanomas [4], and 5.3% of vulvar cancers [8]. The frequency of VM is higher in Caucasian women [9], and typically occurs postmenopause with an average age at diagnosis of 68 years [10,11]; however, it can also affect the pediatric population, and a few such cases have been reported in the literature [12,13]. The majority of VMs are diagnosed at an advanced stage.

In a large series of vulvovaginal melanomas involving 1863 patients, 31.6% had locally advanced or metastatic disease [10]. VM is characterized by a poor prognosis compared to CM [3], with an estimated five-year survival of 47% compared to 92% for CM [4]. This may be attributed to generally delayed diagnosis compared to CM. Clinically, pigmented vulvar lesions account for 12-19%, the majority of which are related to vulvar melanosis [14]. VM presents clinically as a pigmented lesion which may be a macule, papule, or nodular lesion, with irregular borders, and heterogeneous color, occasionally reddish-achromatic (following the ABCDE criteria - A: asymmetry, B: irregular Borders, C: Color heterogeneity, D: Diameter >6 mm, E: Evolution) [15]; other associated symptoms may include pain, bleeding, itching, dysuria, and dyspareunia [16].

VM mostly localizes to the labia majora, labia minora, and clitoris [17]. Clinical examination should involve systematically searching for regional and distant lymphadenopathies as melanoma has a high lymphatic tropism [3]. Histologically, VM constitutes a proliferation of confluent atypical melanocytes with the absence of dermal maturation sometimes associated with cellular necrosis, ulceration, and numerous cutaneous mitoses [15]. Classically, VM expresses the following markers: SOX10, S100 protein, Melan A, and HMB45 (human melanoma black 45) [5]. Several histological subtypes of VM have been described, the most common being lentiginous, nodular, and extensive superficial [10,18]. The molecular profile of VM and melanomas in general differs from CM according to published studies; the c-KIT mutation is the most frequently observed (21.6% of VMs) and this high frequency also helps to distinguish VMs from vaginal melanomas. The second most common mutation encountered is the NRAS mutation (10.2% of VMs), while the BRAF mutation is found in only 8.2% [4,19].

The standard treatment for localized VM is surgical resection while maintaining safety margins according to Breslow thickness of the tumor used for CM: a margin of 0.5-1 cm should be maintained in the case of in situ melanoma; 1 cm for invasive melanomas with thickness ≤1 mm; 1-2 cm when Breslow is 1.01-2 mm; and a margin of 2 cm for a Breslow >2 mm [20,21]. Surgical treatment should also consider the preservation of sexual function and urinary continence [4]. Lymph node dissection is systematic in cases of clinical lymphadenopathy, and sentinel lymph node detection should be proposed in patients with VM without clinical lymphadenopathy or in the presence of ulceration [22].

Radiotherapy may be considered in certain situations, either as neoadjuvant to reduce tumor volume, or as adjuvant depending on prognostic factors, particularly lymph node involvement, tumor size, and quality of tumor resection margins [9]. An unresectable VM has the same prognosis as a metastatic VM. The treatment in this case mainly involves the combination of anti-CTLA-4 (cytotoxic T-lymphocyte antigen 4 protein) and anti-PD1 (programmed cell death-1) immunotherapy, which is a standard first-line treatment such as the combination of ipilimumab/nivolumab or anti-PD1 immunotherapy as monotherapy, which is also a validated option such as pembrolizumab or nivolumab [20]. Beyond first-line treatment, immunotherapy remains the preferred option, but in cases of targetable mutations such as c-KIT (which is frequent), KIT inhibitors such as nilotinib and imatinib may be proposed [20,22].

VM is an uncommon localization of melanoma that is characterized by its poor prognosis due to its location, which significantly affects the quality of life, and also due to its survival rates, which are much lower compared to CMs (an overall estimated five-year survival of 47% vs. 92% for CM) [4]. Hence, multidisciplinary management and inclusion in clinical trials are important for these patients to provide personalized treatment [21].

## Conclusions

VM poses significant challenges in diagnosis and management due to its rarity and aggressive nature. Prompt diagnosis through histopathological and immunohistochemical analysis is crucial, guiding tailored treatment strategies. Surgical resection remains the standard of care for localized disease, while unresectable or metastatic cases require innovative approaches such as radiotherapy and immunotherapy. Multidisciplinary collaboration is essential for optimal patient care. Participation in clinical trials is vital for advancing VM management. Despite its complexity, concerted research efforts offer hope for improved outcomes in VM patients. Additionally, it is imperative to emphasize educating providers regarding pigmented vulvar lesions and their potential differential diagnosis of melanoma, encouraging early biopsy to facilitate timely intervention and thereby enhance patient prognoses.
